# Antibacterial Effect of Spanish Honeys of Different Botanical Origins against *Staphylococcus epidermidis*

**DOI:** 10.3390/ijms25126590

**Published:** 2024-06-15

**Authors:** Vanesa Núñez-Gómez, Marta San Mateo, Lorena Sánchez-Martínez, María Jesús Periago

**Affiliations:** Department of Food Technology, Food Science and Nutrition, Faculty of Veterinary Sciences, Biomedical Research Institute of Murcia (IMIB-Arrixaca-UMU), Regional Campus of International Excellence “Campus Mare Nostrum”, University of Murcia, 30100 Murcia, Spain; vanesa.nunez@um.es (V.N.-G.); marta.sanm@um.es (M.S.M.); lorena.sanchez14@um.es (L.S.-M.)

**Keywords:** honey, antibacterial, (poly)phenol, antioxidant, botanical origin, colour, *Staphylococcus*, wound, infections.

## Abstract

Honey is traditionally used for its medicinal properties attributed to its antibacterial and antioxidant effects. It is considered a natural alternative to conventional antibiotics. This effect has been attributed to their physico-chemical properties, as various chemical parameters can synergistically influence this effect. The aim of this study is to assess Spanish honeys of diverse botanical origins for their antibacterial efficacy against *Staphylococcus epidermidis*, correlating their physico-chemical attributes, (poly)phenol content, and antioxidant activity. The methods included colour determination via two methodologies, acidity, pH, moisture content, and sugar concentration. (Poly)phenol content was quantified using the Folin-Ciocalteau method, while antioxidant activity was evaluated via the FRAP method. Subsequently, the minimum inhibitory concentration (MIC) and minimum bactericidal concentration (MBC) against *S. epidermidis* were investigated with different concentrations of honeys. The results revealed a direct relationship between honey darkness, (poly)phenol concentration, antioxidant activity, and antibacterial efficacy. Darker honeys exhibited higher (poly)phenol levels, greater antioxidant activity, and consequently, lower MIC and MBC values, showing enhanced antibacterial properties. These findings underscore the potential of honey as a therapeutic agent against *S. epidermidis*, particularly in wound healing applications to avoid infection. Further research into honey’s multifaceted properties is warranted to unveil novel therapeutic avenues in healthcare.

## 1. Introduction

Honey is a natural product produced by bees (*Apis mellifera*) from the nectar of plants, which has been used since ancient times for both nutritional and therapeutic purposes [1]. Its primary components encompass soluble carbohydrates (fructose, glucose, and oligosaccharides), water, and minor constituents such as proteins, vitamins, and minerals. The chemical composition of honey is variable, influenced by factors such as botanical origin, climatic conditions, processing methods, handling and storage [1]. The colour of honey is also an important factor, as it is directly related to its composition and botanical origin. Furthermore, the colour determines the price and acceptability to consumers, who tend to prefer lighter honeys, while darker honeys are more appreciated only in certain regions [2]. Additionally, honey contains (poly)phenols, derived from the secondary metabolism of plants. The presence of these compounds is closely linked to the botanical origin of the honey, with flavonoids and phenolic acids being the predominant groups [1,3]. The content of (poly)phenols plays a crucial role in determining the organoleptic properties of honey and serves as a valuable tool for classification and authentication. These compounds are the markers of floral origin in the classification and authentication of honeys [4,5,6]. In addition, (poly)phenols also act as the markers of the biological value of honey, since these compounds exert different biological activities (antioxidant, anti-inflammatory, antibacterial, etc.). Therefore, due to its antioxidant activity, honey can inhibit the formation of free radicals [7]. Moreover, the water-soluble antioxidant fraction contains (poly)phenols such as chrysin, quercetin, kaempferol, galangin, pinobanksin, and pinocembrin, and also catalase (CAT), creating a single antioxidant system. Other compounds involved in the antioxidant effect of honey include ascorbic acid and other natural acids, tocopherols, superoxide dismutase (SOD), reduced glutathione (GSH), Maillard reaction products, and peptides [7], which are linked with the prevention of oxidative stress, inflammation, and several chronic diseases [3,8].

Honey, renowned for its historical application in diverse cultural traditional medicines as a potent antibacterial agent, has seen a resurgence of interest. Despite its diminished role in current medical practices, the escalating resistance of various bacterial strains to antibiotics poses a substantial public health threat [1,9]. Consequently, the imperative for alternative strategies to combat microbial infections has prompted a re-examination of traditional therapeutic remedies, including plant-derived products like honey [10]. The antibacterial efficacy of honey stems from multiple factors, its high sugar content, low pH, hydrogen peroxide generated through glucose-oxidase activity, methylglyoxal, the antimicrobial peptide bee defensin-1, and the presence of (poly)phenols and lysozyme. These components act together to inhibit microbial growth [11,12]. Notably, honey exhibits promising potential in preventing wound infections by fostering wound healing and scar formation [13]. Additionally, certain flavonoids present in honey, such as galangin and chrysin, have demonstrated the ability to inhibit the activity of cyclooxygenase (COX) and lipooxygenase, thereby reducing the expression of cyclooxygenase-2 (COX-2) [14]. This inhibition results in a diminished formation of prostaglandins, consequently mitigating inflammation in the affected tissues [15]. Several studies have evaluated its wound-healing potential. Medical grade honey is a promising treatment for wound healing and can be used as an alternative or complementary treatment to conventional wound infection treatments [16,17]. Therefore, the use of honey and its derivatives in wound healing may be related to their effect on *Staphylococcus epidermidis*. This Gram-positive bacterium belongs to the *Staphylococcaceae* family and is a common coloniser of the skin and mucous membranes of humans and other mammals. They are the most common staphylococcal species in humans: an opportunistic pathogen with high rates of resistance to different classes of antibacterial (including an estimated 70% to 95% of *S. epidermidis* strains being resistant to methicillin) [15]. *S. epidermidis* exhibits a notable proficiency in biofilm formation. While various factors influence the process of wound healing, bacterial infections and the existence of biofilm can exert a substantial impact on this intricate physiological phenomenon. Remarkably, only a limited number of wound care products have undergone assessment for their antibiofilm efficacy, and among these, honey has been a subject of investigation [18,19].

The aim of this work was to evaluate the antibacterial activity of various Spanish honeys from different botanical origins on *S. epidermidis* and to relate it to their physico-chemical properties, their (poly)phenol content, and antioxidant activity.

## 2. Results and Discussion

### 2.1. Composition and Characterisation of Honey Samples

Table 1 shows the mean contents of pH, titratable acidity, moisture, and sugar concentration in the honey samples. Regarding pH, the mean values ranged between 3.9 and 4.4 for the orange blossom and heather honeys, respectively, showing significant differences (*p* < 0.05). However, the eucalyptus and rosemary, polyfloral and thyme honeys showed no significant differences between them. These pH values are within those reported in the scientific literature, as honey is an acidic food, with the pH values ranging from 3.2 to 4.5 depending on the botanical origin [8].

Regarding titratable acidity, the content range varied from 15.5 mEq of acid/kg for the orange blossom honey to 44 mEq of acid/kg for the heather honey, demonstrating significant differences (*p* < 0.05) between these two samples. The polyfloral, eucalyptus, and thyme honeys exhibited no statistically significant differences among themselves. The primary acid found in honey is gluconic acid, which comes from glucose/oxidase activity leading to hydrogen peroxide and gluconic acid, acting as natural preservatives and antibacterial agents [20]. Despite the acid pH and the presence of gluconic acid and other organic acids, the sour taste is not organoleptically perceptible due to the high concentration of sugars. The observed acidity values fall within the reference range outlined in the quality standard according to the maximum allowed established by the Codex Alimentarius (50 mEq of acid/kg of honey) [21]. This effect indicates the absence of fermentation caused by the growth of osmophilic yeasts. These yeasts, such as *Saccharomyces bisporus var. mellis*, *Saccharomyces rouxii*, and *Saccharomyces bailii var. osmophilus*, which may originate from deceased bees, nectar, or soil, have the potential to compromise the hygienic quality of honey [22]. Moreover, it is remarkable that other authors have reported a range between 5.3 and 21 mEq of acid/kg, which is in the range observed in our results [20].

Regarding moisture content, the polyfloral honey sample exhibited the highest value at 21.6%, whereas the eucalyptus honey sample displayed the lowest content at 16%. This disparity between the two samples was statistically significant (*p* < 0.05), but when comparing eucalyptus honey to orange blossom, rosemary, thyme, and heather honeys, no significant differences were observed among them. According to the Codex Alimentarius [21] and the Spanish Legislation (RD 1049/2003) [23], the moisture percentage should not exceed 20%, as honey exceeding this threshold are generally prone to fermentation, resulting in a watery consistency. To reduce the water content, heat treatment, which is prohibited by the Spanish legislation [23], can be applied. However, this approach may result in a decline in the overall honey quality, accompanied by the emergence of undesirable compounds, such as hydroxymethylfurfural—a compound formed by fructose degradation in an acidic medium due to temperature and storage time. Elevated levels of hydroxymethylfurfural are associated with the deterioration of colour, aroma, and flavour [20]. In the case of the polyfloral honey, it was noted that it did not conform to the legislated values, exhibiting a moisture content surpassing 20%. But it should be noted that the sample was not suspected to have fermented based on the acidity and pH values.

According to Molan (1992) [24], honey has a strong osmotic effect, impeding bacterial growth through the potent interaction between sugar and water molecules. The sugar content, measured using a refractometer, was consistently above 80 °Brix for all the samples. The polyfloral honey stood out as the sweetest, while the orange blossom honey contained the lowest sugar percentage, though no significant differences were noted among the samples analysed.

### 2.2. Colour of Honey Samples

**Figure 1:**
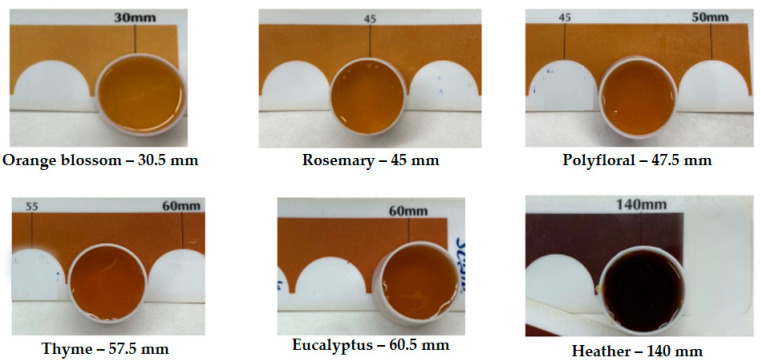
Colour of the honey samples based on the Pfund scale.

The colour of honey is influenced by various factors and holds significant commercial importance, as it directly impacts its pricing. The richness of honey in calcium phosphate and iron increases with its darkness. The globally standardised method for measuring honey colour involves optical comparison using the Pfund scale comparator [25], with the results depicted in Figure 1. This method expresses colour in millimetres, spanning from white to dark brown tones. Analysis revealed that the orange blossom honey exhibited the lightest colour at 30.5 mm, while the heather honey displayed the darkest colour at 140 mm.

Honey colour can also be measured objectively with the reflectance technique using a colorimeter and obtaining the coordinates L*, a*, b*, C*, and hue angle as is shown in Table 2.

The L* parameter ranged between 31.3 and 34.7, with the heather honey sample showing the lowest value as it is the darkest honey, in line with results previously published by other authors [2,26]. The a* parameter reached its highest level in the thyme honey, with a value of 3.2. In contrast, the heather honey showed the lowest value, which was 0.8, meaning that the thyme honey has the most orange-reddish colour of all the honeys analysed. The honeys with the highest b* parameter tend more towards yellowish colours. In this case, it ranged between the values of 4.3 for the eucalyptus honey and 7 for the orange blossom honey, which means that this honey is the most yellowish of all the samples analysed.

The C* value, which is the colour saturation, fluctuated between the values of 7.3 and 2.1 for the orange blossom and heather honeys, respectively. Thus, the heather honey was found to have the least amount of coloured pigments, being the darkest sample. The hue angle ranged from 55° for the eucalyptus honey to 73.7° for the orange blossom honey. Thus, the orange blossom honey has the most yellowish colour and the eucalyptus honey has an orangey colour, tending towards red, with the other honeys showing intermediate values. This tendency was similar to that observed in the Pfund scale comparator.

### 2.3. Total (Poly)phenol Content and Antioxidant Activity

Table 3 displays the total (poly)phenol content and antioxidant activity found in the studied honeys. The phenolic compound levels varied between 315.9 mg and 737.7 mg GAE/kg for the rosemary and heather honeys, respectively. Significant differences were observed among the samples (*p* < 0.05), except for the comparison between the orange blossom and rosemary honeys. The samples with intermediate values, such as polyfloral, eucalyptus, rosemary, and thyme, also exhibited significant differences among them (*p* < 0.05). The literature reveals diverse data on the (poly)phenolic content of honey, influenced by its botanical origin. Dark honeys like buckwheat and molasses honeys typically have total phenolic content (TPC) values around 2 g GAE/kg [27], while light honeys, such as polyfloral and lime blossom honeys, contain few (poly)phenols (295 and 412 mg GAE/kg, respectively) [28]. The content of these compounds has also been studied by other authors in 16 Spanish honeys, showing differences based on the botanical origin [7].

In general, the quantification of total (poly)phenol content in our samples aligns with established ranges in the scientific literature [7,28]. It is imperative to underscore that while the Folin-Ciocalteau assay serves as a prevalent method for ascertaining total (poly)phenol content in food extracts, its lack of specificity for (poly)phenol quantification should be acknowledged. This limitation stems from the capacity of other constituents in honey, such as reducing sugars and amino acids, to reduce the FolinCiocalteau reagent, as noted by Combarros-Fuertes et al. (2019) [7].

The antioxidant capacity was measured by ferric-reducing antioxidant power (FRAP) assay to assess the presence of reducing compounds in honey based on the efficacy of the sample in reducing the Fe^3+^/Fe^2+^ pair. The antioxidant capacity exhibited a range from 192 µmol eq. Trolox/kg for the orange blossom honey to 790 µmol eq. Trolox/kg for thyme honey, thereby manifesting statistically significant differences between these two samples (*p* < 0.05). Conversely, no statistically significant differences were discerned among the orange blossom, polyfloral, eucalyptus, and rosemary honeys, nor between the thyme and heather honeys. In the realm of FRAP values, the botanical origin does not exert a pronounced effect in our samples, as in the case of TPC results. This is because several honey compounds, like (poly)phenols and Maillard compounds, act together to reduce iron, explaining this phenomenon [7].

The results obtained in the present study are similar to those reported by other authors [28,29]. These authors determined the highest FRAP value for dark honey samples, such as carob, arbutus, and eucalyptus honeys, and the lowest FRAP value for light honey, such as citrus honey. The authors indicate that dark honeys showed a higher content of phenolic compounds than light honeys, which is directly related to their higher reducing power [28,29]. These results are in agreement with the results of the TPC and FRAP values in the present study.

### 2.4. Antibacterial Activity

Table 4 shows the MIC and MBC of the different samples, obtained after testing for antibacterial activity against *S. epidermidis*. All the honeys were evaluated, but after testing the rosemary honey the data was inconsistent, so we decided not to include the results of this sample.

The MIC of the studied honeys ranged between 0.1 g/100 mL and 10.6 g/100 mL for the thyme and orange blossom honey, respectively, with significant differences between them (*p* < 0.05). The MBC reached its maximum in the eucalyptus honey, with a value of 30.6 g/100 mL, and its minimum in the heather honey, with a value of 12.4 g/100 mL. Hence, the heather honey was the sample that showed the strongest antibacterial activity, with significant differences between with other three honey samples (*p* < 0.05). For both the MIC and MBC values, no significant differences were observed between the orange blossom, polyfloral, and eucalyptus honeys, nor were they observed when comparing the thyme and heather honeys. These findings indicate that all tested honeys possess the ability to inhibit the growth of *S. epidermidis* and induce its death, but it depends on the botanical origin. In addition, this aligns with previous studies that showed the antibacterial activity of honeys against several bacteria, such as *S. aureus*, *S. epidermidis*, *S. Typhimurium*, *E. coli,* and *P. aeruginosa*. However, the effective concentrations vary depending on the botanical origin as was also depicted in our study [30,31,32].

Swabbing bandaged wounds with honey has shown that infectious bacteria are rapidly wiped out, contributing to the healing process. While antibiotics and antiseptics cause tissue damage, slowing down the healing process, honey is better than modern hydrocolloid dressings as a moist dressing [17,33,34]. In the literature, the antibacterial effect of various types of honeys on different bacterial strains such as *S. epidermidis* and *S. aureus* has been described. These bacteria have developed resistance to many antibiotics and have become the predominant agent of hospital wound sepsis [30,35]. Particularly, Basualdo et al. (2007) [35] reported that 60% of the investigated honeys showed an inhibition of the growth of *S. epidermidis.* However, these authors investigated the MIC in different honeys provided by honey packers and local apiarists, and the samples were classified according to the handling but not by the botanical origins. In addition, these authors used the well/agar diffusion assay and not the dilution method using microplate, and for this reason, we cannot compare our results with those previously reported. They observed that the honey inhibited the growth of *S. epidermidis* when applied undiluted to the cultured plates, whereas no inhibition of the bacterial growth was observed when honey was diluted between 75 and 10%. The study carried out by Morroni et al. (2018) showed that four different honeys (Manuka, African, *Apis mellifera,* and *Melipona beecheii*) have minimum active dilution concentrations of 9, 7, 14, and 1%, respectively, against *S. epidermidis* [19]. Furthermore, these authors showed that a honey concentration above 8% led to a significant reduction in the biomass of established *S. epidermidis* biofilms, which they suggested that the different honeys not only inhibit the growing of this bacteria but also avoid the biofilm formation [19], and hence, *S. epidermidis* exhibited significant susceptibility to honey.

**Figure 2:**
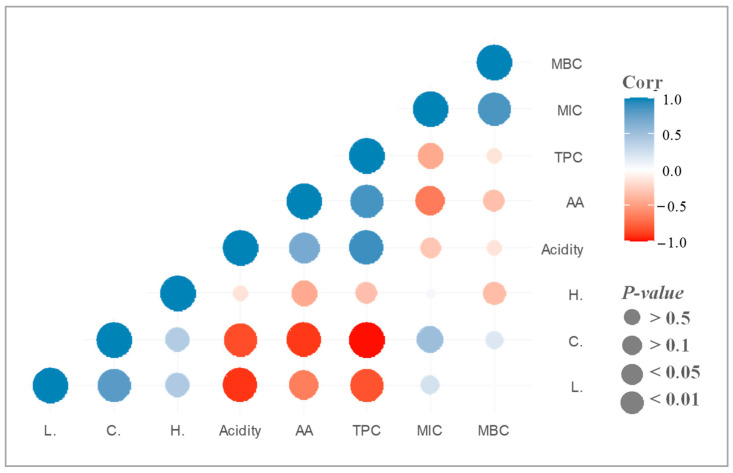
Correlation analysis between all the variables analysed, colour parameters (L*, C*, and H*), acidity, antioxidant activity (AA), total phenolic content (TPC), minimum inhibitory concentration (MIC), and minimum bactericidal concentration (MBC) in the honey samples.

The correlation analysis shown in Figure 2 reveals that the C* parameter was positively correlated with MIC and MBC, indicating that the darkest honeys have a higher potential inhibition of *S. epidermidis*. Moreover, all the colour parameters have a negative correlation with the antioxidant activity and TPC, confirming that (poly)phenols are mainly responsible for the dark colour of honeys. At the same time, the MIC and MBC were negatively correlated with the TPC and antioxidant activity, indicating that these bioactive compounds and their biological activity contribute significantly to the antibacterial activity as has been previously discussed by other authors [28,29]. Hence, a higher C* value or darkness, higher content of TPC and antioxidant activity, and lower values of MIC and MBC, since a lower concentration of honey is necessary to reduce the *S. epidermidis* growth.

Based on the obtained results, we can conclude that the physico-chemical properties of pH, titratable acidity, moisture, and colour parameters depend on the botanical origin of the honeys. There is also great variability in the total (poly)phenol content and antioxidant activity, also depending on the botanical origin. The studied honeys, except the rosemary honey, showed antibacterial activity against *S. epidermidis*, showing inhibitory activity against the growth of the microorganism and bactericidal activity. The findings highlight the antimicrobial and antioxidant properties, as well as the (poly)phenol content of the Spanish honeys. According to our results, for medical and therapeutic uses of honeys, dark samples should be selected since they have the highest (poly)phenol content and consequently the highest antibacterial activity against the *S. epidermidis*. However, conducting additional studies is crucial to enhance our understanding of the specific antibacterial properties of honey and its potential applications in wound healing. Such investigations contribute to optimising hospital management by considering factors like tissue healing, especially in cases where conventional antibiotics may pose a risk of tissue damage.

## 3. Materials and Methods

### 3.1. Samples

For the present study, 6 samples of commercial honeys of different botanical origins were acquired from retailers in the region of Murcia (Spain), classified as follows: orange blossom, polyfloral, eucalyptus, rosemary, thyme, and heather honeys.

### 3.2. Determination of pH and Acidity

The pH was measured in a homogenised solution of honey with distilled water (1:5 *w*/*v*). The pH was then measured with a previously calibrated Crison^®^ pH-Meter Basic 20 pH-meter (Crison Instruments S.A., Barcelona, Spain) immediately after homogenisation in order to avoid honey precipitation [36].

The titratable acidity was determined after an acid/base titration according to the volume of 0.1 N sodium hydroxide necessary to raise the pH of a honey sample dissolved in distilled water (10 g of honey in 75 g of distilled water) to a pH value of 8.3. The titratable acidity is expressed as milliequivalents (mEq)/kg of honey [36].

### 3.3. Moisture Content and °Brix

The indirect refractive index method was used to determine the water content of the honeys using an Abbemat 200 refractometer (Anton Paar, Madrid, Spain) and the tables indicated by Chataway (1932; 1935) [37,38]. With this method, the percentage of moisture in the samples was computed by referencing the correlation table between the refractive index and moisture content, maintained at a constant temperature of 20 °C [23,24]. Additionally, the refractometer facilitated the recording of the total sugar concentration, expressed in °Brix.

### 3.4. Colour Determination by Pfund Scale and CIE Lab Colour Space

For honey, colour depends on several factors and is of great importance from a commercial point of view, as it determines its price. The Pfund scale is a standardised colorimetric technique used both in the farm and in the laboratory for comparative colour determination. This technique categorises the colour of honey according to Pfund millimetres, which are obtained by comparing the colour of the test sample with a previously established table that corresponds to the Pfund scale [39]. This scale is related to the floral origin of the sample and the data are expressed in millimetres [25].

To determine the colour parameters, according to the CIE Lab colour space, the reflectance technique was used. For the measurement, the glass cuvette was filled with honey, and the colour was measured with the Minolta CR Colorimeter (Konica Minolta Sensing Americas, Inc., Ramsey, NJ, USA), expressing the data as coordinates L*, a*, b*, C* and metric hue angle [25]. In this system, the brightness or lightness of the colour is represented by the parameter L* and takes values from 0 (dark) to 100 (light). The parameter a* defines the deviation of the colour towards red, in case the value is positive, or varies towards green in case it is negative, taking values from +50 to −50. The parameter b* defines the deviation of the colour towards yellow, if positive, or towards blue, if negative, and takes values from +50 to −50. The code C* represents the “chroma” (the quantity, purity, or saturation of the colour) and the parameter H corresponds to the “metric angle of hue”, expressed in degrees, on the colour wheel [25].

### 3.5. Total Phenolic Content and Antioxidant Capacity Analysis

The Folin-Ciocalteau method, as outlined by Singleton and Rossi in 1965 [40], was employed to assess the TPC. In the colorimetric assay, 100 µL of each honey sample solution was treated with Na_2_CO_3_ and the Folin-Ciocalteau reagent. Subsequently, a blue chromophore emerged due to the reduction of phosphomolybdic/phosphotungstic complexes, resulting in the formation of tungsten and molybdenum oxides. Following a 1 h incubation period at room temperature, absorbance readings were taken at 750 nm using a UV-visible spectrophotometer (Evolution 300, Thermo-Scientific, Manchester, UK). Gallic acid (Riedelde Haën, Hannover, Germany) served as the standard, and the TPC in the samples was quantified as mg of gallic acid equivalents (GAE)/kg of the sample.

For the assessment of antioxidant capacity, the FRAP assay was employed, following the methodology outlined by Benzie and Strain in 1996 [41]. In short, 100 µL of each honey sample solution were blended with 900 µL of the FRAP reagent. Absorbance readings were taken at 593 nm using a UV-visible spectrophotometer (Evolution 300, Thermo-Scientific, Manchester, UK) precisely 4 min after the initiation of the reaction. The FRAP reagent consisted of 0.3 M acetate buffer, a 10 mM 2,4,6-tripyridyl-s-triazine (TPTZ) solution in a 40 mM HCl solution, and FeCl_3_·6H_2_O solution in the following proportions: 20 mL acetate buffer, 2 mL TPZP, and 2 mL FeCl_3_·6H_2_O. Trolox served as the standard, and the results were expressed as µmol Trolox equivalents (TE)/kg of the sample.

### 3.6. Evaluation of the Antibacterial Activity

The antibacterial activity of honey was evaluated against *S. epidermidis* in a culture medium supplemented with different concentrations of the studied honeys. The broth dilution procedure was followed by performing the microdilution method [30]. It was developed by using 96-well plates to determine the MIC, which is defined as the minimum amount of an antibacterial substance or antibacterial substance that is capable of inhibiting the growth of a microorganism under standardised conditions. In addition, the MBC was also calculated, which aims to determine the lowest concentration of an antibacterial that is capable of killing 99.9% of the initial bacterial strain [42].

First, the culture media to be used in the assay were prepared using the Mueller/Hinton (MH) broth (Oxoid, Hampshire, UK), which was used together with tryptone soya agar (Oxoid, Hampshire, UK), as they facilitate the growth of different bacterial strains. To prepare the honey samples, the protocol described by McLoone et al. (2021) was followed with some modifications [43]. In short, 25 g of honey was diluted in 50 mL of MH (*w*/*v*) and filtered through a 0.45 µm pore size filter. From this stock dilution, the rest of the working dilutions were prepared with MH broth, which were different according to the type of honey, and whose concentrations ranged from 1.25 to 20 g/100 mL, as shown in Table 5.

For inoculum preparation, *S. epidermidis* was isolated on Baird/Paker selective medium (Oxoid, Hampshire, UK) from a skin sample. It was incubated on Brain Heart Infusion Agar (BHI) (Scharlau, Valencia, Spain) and identified using an API gallery (Api Staph V5.0, Biomérieux España S.A., Madrid, Spain) with a 97.5% match, maintaining the inoculum on BHI agar weekly during the experiment period. Prior to the antibacterial activity assays, the bacterial concentration with the McFarland scale using the DENSIMAT densitometer (Biomérieux España S.A., Madrid, Spain) was determined. For the preparation of the reading plate, dilutions in MH broth and in the working honey dilutions were carried out until a concentration of 3 × 10^5^ cfu/mL was reached [44].

The microdilution method described by Green et al. (2020) was followed with some modifications [44]. First, to evaluate the growth of *S. epidermidis*, 200 µL were added to each well of the microplate, preparing different rows: a control well (corresponding to the culture medium together with the inoculum, without the honey sample), a sample blank well (representing the working dilutions of each honey sample to correct the absorbance according to the colour of the sample) and experimental sample wells (corresponding to the different dilutions of the honey samples together with the inoculum). The absorbance was measured in a microplate spectrophotometer (BioTek Instruments, Winooski, VT, USA) at 600 nm every hour, for 20 h at 37 °C, shaking every 15 min. Each sample was repeated 3 times, the percentages of growth of *S. epidermidis* in the different dilutions were calculated, and the equations relating concentration and growth were obtained. From these equations, the MIC and MBC of the different studied honeys were calculated.

### 3.7. Statistical Analysis

The data underwent processing through R Studio version 4.0.5., developed by the R Foundation for Statistical Computing in Vienna, Austria. Each assay was carried out in triplicate to ensure reliability. Normality was assessed using the Shapiro/Wilk test, while the homogeneity of variances was determined using the Bartlett test. To identify significant differences at a *p*-value < 0.05, the one-way analysis of variance (ANOVA) was used using Tukey’s test as a post hoc test. Correlation analyses were performed using the Pearson correlation test to evaluate the relationship between the studied variables.

## Figures and Tables

**Table 1:** pH, acidity (mEq of acid/kg), moisture (%), and sugar concentration (°Brix) measured in honey samples ^1^.

Honey Sample	pH	Acidity	Moisture	Sugar Concentration
Orange blossom	3.9 ± 0.3 ^d^	15.5 ± 0.7 ^c^	17.5 ± 0.1 ^b^	80.7 ± 1.0
Polyfloral	4.0 ± 0.0 ^c^	22.8 ± 2.5 ^b^	21.6 ± 0.9 ^a^	82.6 ± 0.0
Eucalyptus	4.2 ± 0.0 ^b^	22.8 ± 0.4 ^b^	16.0 ± 0.3 ^b^	82.1 ± 0.1
Rosemary	4.1 ± 0.0 ^b^	16.0 ± 0.7 ^c^	16.1 ± 0.1 ^b^	82.1 ± 0.1
Thyme	4.0 ± 0.0 ^c^	25.8 ± 0.4 ^b^	16.4 ± 0.3 ^b^	81.9 ± 0.2
Heather	4.4 ± 0.0 ^a^	44.0 ± 1.4 ^a^	16.2 ± 0.0 ^b^	81.9 ± 0.0

^1^ values are expressed as mean ± SD (*n* = 3). Different letters a–d indicate significant differences (*p* < 0.05) among the samples.

**Table 2:** Colour parameters measured in honey samples ^1^.

Honey Sample	L*	a*	b*	C*	H*
Orange blossom	34.7 ± 0.0 ^a^	2.1 ± 0.0 ^d^	7.0 ± 0.0 ^a^	7.3 ± 0.0 ^a^	73.7 ± 0.1 ^a^
Polyfloral	33.2 ± 0.8 ^cd^	3.2 ± 0.1 ^a^	6.0 ± 0.1 ^c^	6.8 ± 0.1 ^b^	61.9 ± 0.3 ^d^
Eucalyptus	32.7 ± 0.0 ^d^	3.0 ± 0.1 ^b^	4.3 ± 0.1 ^e^	5.2 ± 0.1 ^c^	55.0 ± 0.2 ^f^
Rosemary	34.3 ± 0.1 ^ab^	2.4 ± 0.0 ^c^	6.5 ± 0.0 ^b^	6.9 ± 0.0 ^b^	70.0 ± 0.3 ^b^
Thyme	33.1 ± 0.0 ^bc^	3.2 ± 0.0 ^a^	4.9 ± 0.0 ^d^	2.7 ± 0.2 ^d^	57.0 ± 0.1 ^e^
Heather	31.3 ± 0.0 ^e^	0.8 ± 0.0 ^e^	1.9 ± 0.0 ^f^	2.1 ± 0.0 ^e^	67.6 ± 0.2 ^c^

^1^ values are expressed as mean ± SD (*n* = 3). Different letters a–f indicate significant differences (*p* < 0.05) among the samples.

**Table 3:** Total (poly)phenol content (mg GAE/kg) and antioxidant activity (µmol Trolox Eq./kg) measured in honey samples ^1^.

Honey Sample	TPC	Antioxidant Capacity
Orange blossom	334.8 ± 14.5 ^e^	191.5 ± 6.7 ^b^
Polyfloral	394.0 ± 8.4 ^d^	334.4 ± 13.8 ^b^
Eucalyptus	439.6 ± 6.9 ^c^	413.0 ± 21.6 ^b^
Rosemary	315.9 ± 6.2 ^e^	245.3 ± 17.2 ^b^
Thyme	651.8 ± 2.2 ^b^	789.5 ± 68.7 ^a^
Heather	737.7 ± 22.9 ^a^	702.2 ± 9.4 ^a^

^1^ values are expressed as mean ± SD (*n* = 3). Different letters a–e indicate significant differences (*p* < 0.05) among the samples.

**Table 4:** Minimum inhibitory concentration (MIC) and minimum bactericidal concentration (MBC) (g/100 mL) measured in the honey samples ^1^.

Honey Sample	MIC	MBC
Orange blossom	10.6 ± 2.4 ^a^	28.0 ± 2.1 ^a^
Polyfloral	7.8 ± 1.4 ^a^	26.8 ± 1.5 ^a^
Eucalyptus	8.6 ± 1.1 ^a^	30.6 ± 4.3 ^a^
Thyme	0.1 ± 0.0 ^b^	16.2 ± 0.5 ^b^
Heather	2.4 ± 1.0 ^b^	12.4 ± 0.8 ^b^

^1^ values are expressed as mean ± SD (*n* = 3). Different letters a and b indicate significant differences (*p* < 0.05) among the samples.

**Table 5:** Concentration of different honeys expressed as mg/100 mL tested in the antibacterial assay.

Honey Sample	1.25	2.5	5	6.25	7.5	10	15	20
Orange blossom		X			X	X	X	X
Polyfloral		X			X	X	X	X
Eucalyptus		X			X	X	X	X
Rosemary	X	X			X	X	X	
Thyme	X	X			X	X	X	
Heather	X	X	X	X	X			

## Data Availability

Data is contained within the article.
